# Anemia impacts short-term and long-term mortality risk after acute ischemic stroke

**DOI:** 10.3389/fstro.2026.1859301

**Published:** 2026-07-28

**Authors:** Chen Ee Low, Hon Jen Wong, Chun En Yau, Jia Yang Tan, Yao Neng Teo, Yao Hao Teo, Jasper R. Senff, Reinier Tack, Andrew F. W. Ho, Bernard P. L. Chan, Leonard L. L. Yeo, Joshua Y. P. Yeo, Ching-Hui Sia, Benjamin Y. Q. Tan

**Affiliations:** 1 Department of Medicine, Yong Loo Lin School of Medicine, National University of Singapore, Singapore, Singapore; 2 Department of Neurology, Massachusetts General Hospital, Boston, MA, United States; 3 Department of Neurology, University Medical Center Utrecht, Utrecht, Netherlands; 4 Department of Emergency Medicine, Singapore General Hospital, Singapore, Singapore; 5 Pre-Hospital and Emergency Research Centre, Duke-National University of Singapore Medical School, Singapore, Singapore; 6 Division of Neurology, Department of Medicine, National University Hospital, Singapore, Singapore; 7 Department of Cardiology, National University Heart Centre, Singapore, Singapore

**Keywords:** anemia, ischemic stroke, mortality, polycythemia, stroke

## Abstract

**Background:**

The relationship between hemoglobin concentration and all-cause mortality in acute ischemic stroke (AIS) patients remains unclear due to conflicting findings. This study aims to evaluate the impact of hemoglobin concentration on the risk of both short-term and long-term all-cause mortality in AIS patients from a nationwide stroke registry.

**Method:**

We utilized data from the Singapore Stroke Registry (SSR), including patients >18years old at first AIS onset, and received treatment at a local hospital between 2005 and 2020. Patients were stratified according to sex-specific hemoglobin concentration cut-offs on anemia severity and polycythemia from the World Health Organization. Multivariate logistic regression and Cox proportional hazards modeling were performed, adjusting for demographics and cardiovascular risk factors.

**Results:**

A total of 56,884 ischemic stroke patients were included, of which 66.8% had no anemia, 15.7% had mild anemia, 12.2% had severe anemia, and 5.3% had polycythemia. The cohort was predominantly male (57%), with a median age of 68 years. The median follow-up duration was 10-years (IQR 7–13). Compared to patients with normal-range hemoglobin, both mild and severe anemia was associated with elevated short-term and long-term risks of all-cause mortality. Severe anemia was associated with the highest risk of all-cause mortality, with hazard ratios of 1.99 (95%CI:1.92–2.06) at 30-days, 1.73 (95%CI:1.66–1.81) at 1-year, and 1.52 (95%CI:1.41–1.64) at 5-years. However, the excess risk associated with severe anemia appeared to attenuate at 10-years, whilst the elevated risk persisted among patients with mild anemia.

**Conclusion:**

In this nationwide stroke registry, anemia is prevalent among AIS patients and is significantly associated with both short-term and long-term mortality.

## Introduction

Acute Ischemic Stroke (AIS) is one of the leading causes of mortality worldwide and remains a global public health challenge (Feigin, 2021). According to the Global Burden of Disease (GBD) estimates, there were approximately 12.2 million cases of stroke and 6.6 million deaths globally in 2019 (Global Burden of Disease, 2023). Despite advancements in acute reperfusion therapy and secondary prevention strategies, the burden of AIS remains high due to its severe impact on the patients' quality of life (Chohan and How, 2019). Therefore, it is crucial to understand key prognostic factors that can influence both short-term and long-term all-cause mortality in AIS.

Previous studies have indicated that hematological parameters, particularly blood hemoglobin level, may serve as a prognostic biomarker for AIS outcomes (Barlas et al., 2016). Anemia, characterized by reduced hemoglobin levels, affects 32.9% of the global population (Kassebaum et al., 2014), and is present in approximately 30% of AIS patients according to the hospital-based studies (Barlas et al., 2016). While the association between anemia and increased mortality in chronic diseases such as acute coronary syndrome (Wester et al., 2019), heart failure (Köseoglu and Özlek, 2024) and chronic kidney disease (Portolés et al., 2021) is well-established, the association between anemia and mortality remains unclear in AIS due to conflicting results, and heterogeneity in outcome definitions. For instance, an early study by Bhatia et al. (2004) conducted in Lucknow, India found that hemoglobin levels did not predict 30-day mortality in stroke patients. However, more recent studies, such as that by Tanne et al. (2010) have shown that both high and low extremes of hemoglobin levels are associated with increased mortality rates. Previous studies had significant limitations in their study design, including small (Bhatia et al., 2004; Kubo et al., 2017; Sharma et al., 2018; Tian et al., 2021), single-center cohorts and a focus on short-term outcomes rather than the long-term impact of hemoglobin levels on mortality. For example, Bhatia et al. (2004) only assessed mortality up to 30 days, while Tian et al. (2021) examined outcomes up to 1 year. The influence of polycythemia on long-term stroke mortality also remains unclear due to inconsistent findings across studies.

Blood transfusion strategies for anemia in other acute conditions, such as acute myocardial infarction (AMI), have also been explored, though they remain a topic of debate without standardized guidelines. A recent meta-analysis conducted by Braïk et al. (2024) comparing the clinical outcomes of AMI patients with anemia treated using a restrictive blood transfusion strategy vs. a liberal blood transfusion strategy found no significant difference in patient mortality. Similarly, in critically ill patients with traumatic brain injury and anemia, a liberal transfusion strategy did not reduce the risk of an unfavorable neurologic outcome at 6 months (Turgeon et al., 2024).

Given these gaps, our study aims to evaluate the impact of hemoglobin concentration on both short- and long-term mortality following AIS using a large national stroke registry with follow-up extending up to 10 years. By clarifying these relationships, our findings will provide critical insights to inform the design of future randomized controlled trials assessing blood transfusion strategies in AIS management.

## Methods

### Study population

This study utilized data from the Singapore Stroke Registry (SSR), a prospectively maintained registry of all stroke cases in Singapore (Venketasubramanian et al., 2015). Details regarding the conduct of the registry have been previously described. Briefly, the SSR receives stroke case notifications from all public hospitals *via* inpatient discharge summaries, medical claim listings, and the national death registry, from which data such as demographics, risk factors, stroke subtypes, and outcomes are collected. All notified cases are screened by the registry coordinators to confirm that there was a diagnosis of stroke by a medical practitioner. Yearly audits on the data are performed by a separate team to ensure a data accuracy of more than 95%. Specifically for this study, patients were included if they (1) were >18 years old at first AIS onset, and (2) diagnosed with AIS at a public hospital in Singapore between 2005 and 2020. The local ethics committee approved the study protocol and granted a waiver of informed consent, as the data were de-identified (National Healthcare Group IRB Reference No: 2023/00263). This study was performed in accordance with the Declaration of Helsinki.

### Exposure and outcomes

The exposure analyzed in this study was hemoglobin (Hb) concentration by sex-specific cut-off levels for anemia and polycythemia. The exposure was measured at diagnosis of ischemic stroke. The World Health Organization (WHO) hemoglobin concentration definitions for the assessment of anemia severity and polycythemia were used in our study (WHO, 2011). Normal hemoglobin was defined as 13g/dL ≤ Hb ≤ 16.5g/dL for males and 12g/dL ≤ Hb ≤ 16g/dL for females. Mild anemia was defined as 11g/dL ≤ Hb < 13g/dL for males and 11g/dL ≤ Hb < 12g/dL for females. Severe anemia was defined as Hb < 11g/dL for both sexes. Polycythemia was defined as Hb>16.5g/dL for males and Hb>16g/dL for females (Tefferi and Barbui, 2023). The outcomes measured were 30-day, 1-year, 5-year, and 10-year all-cause mortality. A Kaplan-Meier curve for overall mortality stratified by the different hemoglobin concentration levels was constructed.

### Statistical analysis

Data for continuous variables were reported as median and interquartile range (IQR), while data for categorical variables were reported as count numbers and proportion. Univariate analyses evaluating the association between age group and characteristics of the study population were performed using Pearson's chi-squared test. The Kruskal Wallis test was used in comparison for median ages. The median follow-up time was derived using the reverse Kaplan-Meier method. Time-to-mortality data were graphically represented using the Kaplan-Meier method. A *p*-value of lower than 0.05 was considered statistically significant.

Multivariate analyses were conducted to assess the association between hemoglobin concentration and outcomes using logistic regression for 30-day mortality and Cox proportional hazards models for 1-year, 5-year, and 10-year mortality, with results reported as hazard ratios (HRs). Multivariable models were derived from variables with a *p*-value ≤ 0.1 for no anemia vs. severe in the univariate Cox analysis and after discussion with experts on the clinically relevant factors. The variables adjusted for were demographic factors (sex, race), key cardiovascular risk factors for acute ischemic stroke (hypertension, diabetes, hyperlipidemia, atrial fibrillation, and smoking status) and previous stroke or ischemic heart disease. Additionally, univariate Cox regression analyses with restricted cubic splines were performed to explore potential non-linear relationships between hemoglobin levels and mortality over time. The restricted cubic spline function, optimized using the Akaike Information Criterion and fitted with five knots, was used to estimate survival probabilities across the full range of hemoglobin values.

## Results

### Baseline characteristics of the study population

The baseline sociodemographic characteristics of the study population are presented in Table 1. The study included 56,884 ischemic stroke patients, of whom 66.8% (*N* = 37,993) had normal hemoglobin levels, 15.7% (*N* = 8,917) had mild anemia, 12.2% (*N* = 6,973) had severe anemia, and 5.3% (*N* = 3,001) had polycythemia. The median age of the cohort was 68 years [interquartile range (IQR): 58–78], with patients in the severe anemia group being the oldest, with a median age of 75 years (IQR: 65–83). The cohort was predominantly male (*N* = 32,331; 57%) and of Chinese ethnicity (*N* = 43,361; 76%). The study population had a median follow-up duration of 10 years (IQR: 7–13).

**Table 1:** Characteristics of the study population.

Characteristic	Overall, *N* = 56,884	No anemia, *N* = 37,993	Mild anemia, *N* = 8,917	Severe anemia, *N* = 6,973	Polycythemia, *N* = 3,001
Median age^1^ (IQR)	68 (58, 78)	67 (57, 76)	74 (65, 82)	75 (65, 83)	59 (52, 68)
Gender					
Female	24,553 (43%)	16,130 (42%)	3,653 (41%)	4,382 (63%)	388 (13%)
Male	32,331 (57%)	21,863 (58%)	5,264 (59%)	2,591 (37%)	2,613 (87%)
Ethnicity					
Chinese	43,361 (76%)	29,123 (77%)	6,713 (75%)	5,238 (75%)	2,287 (76%)
Indian	3,706 (7%)	2,481 (6%)	713 (8%)	512 (7%)	221 (7.4%)
Malay	8,089 (14%)	5,675 (15%)	1,337 (15%)	1,077 (15%)	435 (14%)
Others	1,014 (3%)	714 (2%)	154 (2%)	146 (3%)	58 (1.9%)
Current/former smoker	13,884 (24%)	11,070 (29%)	1,918 (22%)	896 (13%)	1,519 (51%)
Hypertension	43,570 (77%)	30,176 (79%)	7,469 (84%)	5,925 (85%)	2,397 (80%)
Diabetes mellitus	23,680 (42%)	15,657 (41%)	4,302 (48%)	3,721 (53%)	1,264 (42%)
Hyperlipidemia	46,661 (82%)	33,889 (89%)	7,493 (84%)	5,279 (76%)	2,674 (89%)
Atrial fibrillation	10,324 (18%)	6,504 (17%)	2,049 (23%)	1,771 (25%)	438 (15%)
Previous stroke/TIA	1,862 (3%)	1,392 (3.7%)	379 (4.3%)	91 (3.0%)	260 (3.7%)
Ischemic heart disease	10,096 (18%)	6,932 (18%)	2,719 (30%)	445 (15%)	2,499 (36%)
Follow-up time in years^2^			9.80 (6.75;13.23)		

^1^Kruskal-Wallis rank sum test, ^2^Median (IQR) 9.80 (6.75; 13.23).

Patients with severe anemia had the highest prevalence of cerebrovascular risk factors, including hypertension (*N* = 5,925; 85%), diabetes mellitus (*N* = 3,721; 53%), and atrial fibrillation (*N* = 1,771; 25%). In contrast, patients with mild anemia had the highest prevalence of previous stroke or transient ischemic attack (TIA; *N* = 379; 4.3%). The prevalence of smoking was lowest in the severe anemia group (*N* = 896; 13%) compared to mild anemia (*N* = 1918; 22%), no anemia (*N* = 11,070; 29%), and polycythemia (*N* = 1519; 51%). Patients with polycythemia were the youngest group, with a median age of 59 years (IQR: 52–68), and had the highest prevalence of ischemic heart disease (*N* = 2,499; 36%) and smoking (*N* = 1,519; 51%).

### Association between hemoglobin concentration category and all-cause mortality

**Figure 1:**
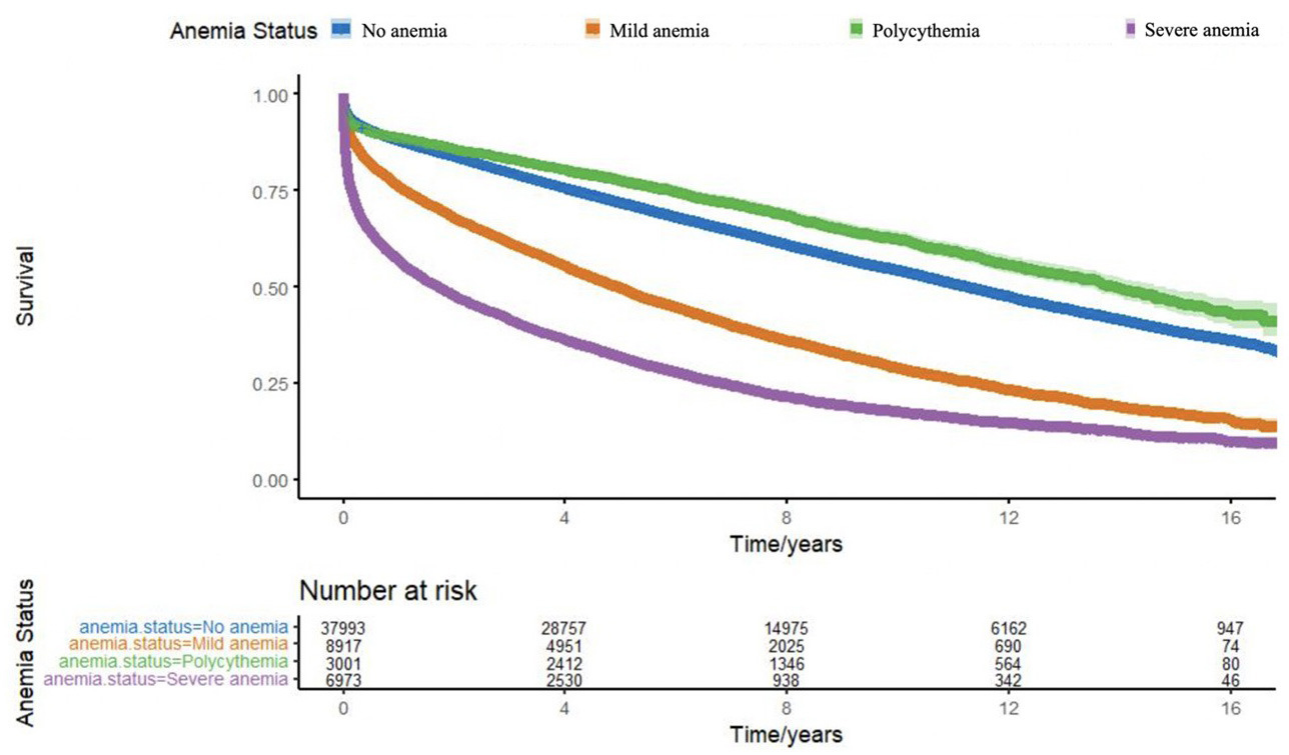
Kaplan-Meier curve for all-cause mortality.

The multivariable analysis of the outcomes is presented in Table 2. Acute ischemic stroke patients with severe anemia had the highest risk of all-cause mortality, with significant hazard ratios (HRs) at 30 days (HR = 1.99, 95% CI: 1.92–2.06), 1 year (HR = 1.73, 95% CI: 1.66–1.81), and 5 years (HR = 1.52, 95% CI: 1.41–1.64). However, the risk became insignificant at 10 years (HR = 1.13, 95% CI: 0.95–1.34). Patients with mild anemia also exhibited a significantly increased risk of all-cause mortality at 30 days (HR = 1.38, 95% CI: 1.34–1.42), 1 year (HR = 1.33, 95% CI: 1.29–1.38), 5 years (HR = 1.28, 95% CI: 1.21–1.35), and 10 years (HR = 1.16, 95% CI: 1.04–1.31). In contrast, AIS patients with polycythemia did not have a significantly increased risk of all-cause mortality at any time point. The Kaplan-Meier curve further supports these findings, demonstrating significantly worse survival for AIS patients with anemia (*p* < 0.001; Figure 1).

**Table 2:** Regression models evaluating the association between hemoglobin concentration and outcomes.

Outcomes	Events/Patients	Hemoglobin concentration			
		No anemia	Mild anemia	Severe anemia	Polycythemia
*30-day mortality*	25,520/52,324	Reference			
Model 1; HR (95% CI)			2.06 (2.00–2.13), *p* < 0.01	3.01 (2.91–3.12), *p* < 0.01	0.74 (0.69–0.79), *p* < 0.01
Model 2; HR (95% CI)			1.38 (1.34–1.42), *p* < 0.01	1.99 (1.92–2.06), *p* < 0.01	1.01 (0.94–1.08), *p* < 0.01
*1-year mortality*	20,006/46,809				
Model 1; HR (95% CI)			1.98 (1.91–2.05), *p* < 0.01	2.51 (2.41–2.62), *p* < 0.01	0.74 (0.69–0.79), *p* < 0.01
Model 2; HR (95% CI)			1.33 (1.29–1.38), *p* < 0.01	1.73 (1.66–1.81), *p* < 0.01	1.00 (0.93–1.08), *p* = 0.91
*5-year mortality*	9,636/32,933				
Model 1; HR (95% CI)			1.84 (1.74–1.94), *p* < 0.01	1.99 (1.85–2.14), *p* < 0.01	0.80 (0.73–0.88), *p* < 0.01
Model 2; HR (95% CI)			1.28 (1.21–1.35), *p* < 0.01	1.52 (1.41–1.64), *p* < 0.01	1.05 (0.96–1.16), *p* = 0.30
*10-year mortality*	2,681/12,817				
Model 1; HR (95% CI)			1.57 (1.40–1.76), *p* < 0.01	1.33 (1.13–1.58), *p* < 0.01	0.86 (0.73–1.01), *p* < 0.01
Model 2; HR (95% CI)			1.16 (1.04–1.31), *p* = 0.01	1.13 (0.95–1.34), *p* = 0.20	1.06 (0.90–1.25), *p* = 0.50

Model 1: univariate.

Model 2: model 1 + demographics and cardiovascular risk factors, specifically admission age, sex, race, hypertension, diabetes, hyperlipidemia, atrial fibrillation, previous stroke/TIA, ischemic heart disease and smoking status.

HR, hazard ratio; CI, confidence interval.

### Restricted cubic spline analysis

**Figure 2:**
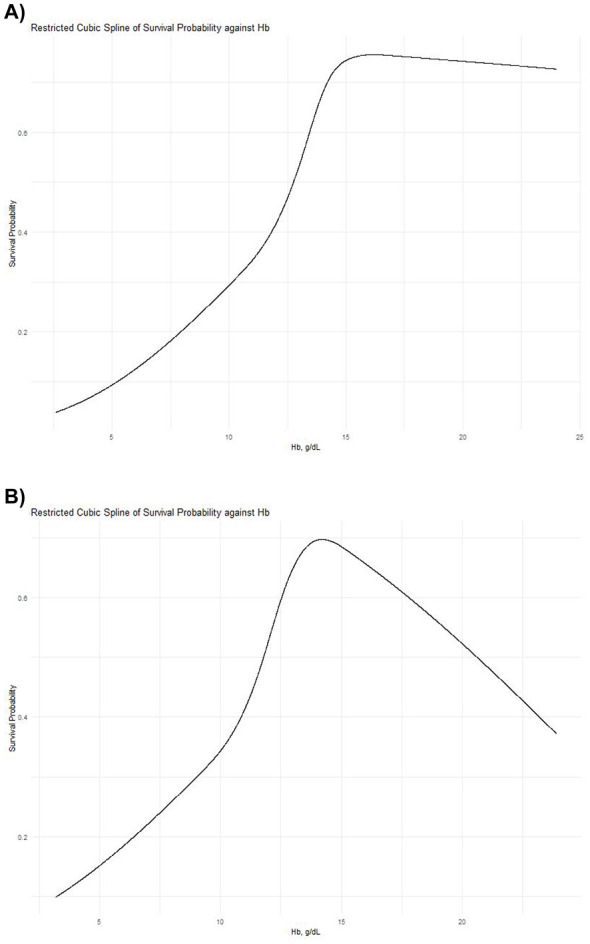
Restricted cubic spline analyses of male **(A)** and female **(B)**.

The restricted cubic spline analyses of survival probability in relation to hemoglobin levels are illustrated in Figure 2. For both males (A) and females (B), survival probability increases with hemoglobin levels up to approximately 15 g/dL. Beyond this threshold, survival probability begins to decline, indicating an elevated risk associated with both low and high hemoglobin levels. Notably, the decline is more pronounced in females than in males, suggesting that females face a higher risk.

## Discussion

In this national registry study of 56,884 ischemic stroke patients, we observed a strong association between anemia and all-cause mortality in AIS patients. Those with severe anemia faced the highest significant risk of all-cause mortality at 30 days, 1 year, and 5 years, but this risk became insignificant at 10 years. Conversely, polycythemia showed no significant impact on mortality risk across any timepoints.

Several pathological mechanisms have been proposed to explain the association between anemia and increased mortality, particularly in the context of AIS. Firstly, the decreased oxygen-carrying capacity of blood exacerbates hypoxia in the ischemic penumbra (Adhami et al., 2006), leading to larger unsalvageable brain tissue and hence worse outcomes (Yan et al., 2011). Secondly, cerebrovascular autoregulation can also be compromised in the setting of AIS. When hemoglobin levels are low, the human body naturally compensates by increasing cardiac output, heart rate and vasodilatory effects on cerebral arteries, ultimately increasing cerebral blood flow (Bateman and Bateman, 2024) to maintain the oxygen transport capacity to brain tissue. However, some studies have reported that in AIS patients with anemia, this autoregulatory effect is impaired, even after reperfusion is achieved through treatment (Inui et al., 2023). This impairment can compromise tissue perfusion, leading to poorer outcomes. Thirdly, anemia has also been suggested to trigger an inflammatory response (Nagel et al., 1994), upregulating the production of chemokines (McLaren et al., 2007) and interleukins (Ferrucci et al., 2005), which in turn propagate cerebral infarction. Furthermore, research on the relationship between anemia and collateral circulation in AIS is still evolving. Collateral circulation plays a crucial role in maintaining blood flow to tissue normally supplied by an occluded vessel during an AIS episode (Maguida and Shuaib, 2023). While recent studies have shown no association between hemoglobin levels and cerebral collateral status, there is evidence of increased coronary collaterals in the context of low hemoglobin levels, likely due to multiple mechanisms (Ramos et al., 2024; Scheel, 1990). Further longitudinal studies exploring how varying hemoglobin levels influence the formation and maintenance of cerebral collaterals could offer valuable insights into improving AIS management.

The excess risk of all-cause mortality with severe anemia was observed to attenuate at 10-years, while elevated risk persisted in patients with mild anemia. This may partly reflect survivorship bias, where those patients with severe anemia who survived to later timepoints represented a specific subgroup already at lower baseline risk. This subgroup may have included patients with more acute, correctable causes of anemia, such as bleeding, for whom early correction of hemoglobin levels may have attenuated longer-term excess risk (Hess et al., 2023). Conversely, patients with severe anemia due to advanced disease, infection or malignancy, may have had higher baseline risk (Gaspar et al., 2015). In addition, severe anemia may have prompted greater clinical attention, diagnostic evaluation, and treatment, whereas mild anemia may have been more likely to remain untreated (Hess et al., 2023), potentially contributing to the persistence of excess long-term risk. Regardless, these findings highlight the need to consider anemia as a dynamic, longitudinal clinical factor post-AIS, rather than a single baseline measurement. Post-AIS anemia-associated risk profiles should ultimately be interpreted according to the individual patient profile.

We also found that ischemic stroke patients with polycythemia did not have any significantly increased mortality risk throughout the different timepoints. The results of polycythemia on post-stroke mortality risk based on the current literature are largely inconsistent. Some studies have found that high levels of hemoglobin levels did increase post-stroke mortality risk. For example, in Sico et al. (2018), patients with polycythemia were found to have an increased risk of in-patient mortality compared to patients with lower hemoglobin levels. A systematic review and meta-analysis conducted by Barlas et al. (2016) also found that elevated hemoglobin levels were associated with increased mortality, especially in the 1st month after an AIS episode. The prevailing hypothesis is that the increased blood viscosity can exacerbate stasis of blood flow and therefore enhance the thrombotic process, reducing reperfusion and salvageable penumbral tissue (Allport et al., 2005). The lack of significant association found in our study could highlight the possible protective factor of increased oxygen-carrying capacity in higher hemoglobin levels enhancing oxygen delivery to brain tissue (Ookawara et al., 2020). The restricted cubic-spline analyses suggests that beyond the hemoglobin level threshold of approximately 15 g/dL, females may have a higher mortality risk compared to males. This sex-specific difference could be attributed to a few possible mechanisms. Firstly, studies (Derham and Buchan, 1989) have shown that higher levels of estrogen and progesterone causes a rise in blood viscosity by raising hematocrit, creating a more thrombogenic environment as explained above. Secondly, the smaller diameter blood vessels in females (Zaid et al., 2023) could mean a larger reduction in blood flow given the same increase in blood viscosity compared to males, thus leading to a higher thrombotic risk. However, our findings and proposed mechanisms should be interpreted as exploratory and hypothesis-generating, as they could not be comprehensively evaluated in this study. They were also not supported by a significant association between polycythemia and mortality in our primary analysis. We also found that patients with polycythemia were younger despite higher rates of smoking and ischemic heart disease, raising the possibility of confounding. Therefore, the potential mortality risk associated with polycythemia after AIS should be interpreted cautiously and considered in the context of individual patient characteristics and risk profiles.

The current American Heart Association guidelines for AIS management recommend that hemodilution offers no benefit and high-dose albumin is ineffective. However, these guidelines fail to specify optimal hemoglobin targets for the AIS population, highlighting a critical gap in the development of tailored treatment strategies (Powers et al., 2019). Our study findings aim to help guide the design of randomized controlled trials assessing blood transfusion strategies in AIS management. Landmark studies such as the TRAIN trial (Taccone et al., 2024) and REALITY trial (Ducrocq et al., 2021) that aimed to assess the most appropriate blood transfusion strategies in the context of acute myocardial infarction patients with anemia can provide us a framework for future clinical trials in an AIS population. Future studies can be conducted to investigate if there is an optimal threshold for initiating blood transfusion in AIS patients with anemia, as well as how different blood transfusion strategies will influence functional and mortality outcomes in this patient population.

On a separate note, anemia across its severity spectrum should not necessarily be considered in isolation, but may instead reflect a broader systemic risk profile. For example, the HALP score, which incorporates hemoglobin, albumin, lymphocyte count, and platelet count as a composite marker of inflammatory and nutritional status, has been associated with prolonged hospital stay, need for intensive care, and mortality in a cohort of 1128 patients with AIS (Umit et al., 2026). Accordingly, anemia identified at AIS presentation should prompt consideration of the patient's broader clinical, disease, and nutritional status, supporting a more holistic approach to post-AIS evaluation and care.

### Strengths and limitations

There are several strengths of our study. Firstly, our large sample size of 56,884 patients from a nationwide registry provides high statistical power to enhance the applicability of our findings. Secondly, our long follow-up duration of up to 10 years allows us to assess both the short-term and long-term mortality risk of AIS patients, compared to other studies that mainly assessed only short-term outcomes. Lastly, the clinical data was linked with mortality records from the Registry of Births and Deaths, which captures all mortality outcomes in Singapore through mandatory reporting, minimizing loss to follow-up and reducing selection bias.

There are several limitations to our study. First, due to its retrospective design, we could only report associations rather than establish causality. Second, the absence of longitudinal hemoglobin trends and corresponding management strategies including fluid administration or blood transfusion introduces the possibility of treatment-related confounding. In addition, a single baseline hemoglobin measurement is unlikely to fully reflect longer-term risk over follow-up extending to 10-years post-AIS. Hemoglobin levels should be considered as a varying clinical marker that changes in response to evolving patient characteristics, underlying conditions and treatment. Third, the underlying etiology of anemia was not captured and may have represented an important source of residual confounding. Low hemoglobin levels at presentation can reflect either an acute process such as bleeding, or a more chronic systemic condition such as chronic kidney disease, autoimmune disease, infection, malignancy, and malnutrition (Hess et al., 2023). These etiologies each carry distinct biological mechanisms, prognostic implications and treatment strategies. Future studies should therefore seek to characterize anemia etiology to better define its association with outcomes after AIS. Fourth, the SSR lacked information on subjective measures of stroke severity, such as the National Institutes of Health Stroke Scale, and objective markers such as large- or small-vessel occlusion, reperfusion status, and need for ICU measurement. Hence, the residual confounding of our results by stroke severity cannot be excluded, given that stroke severity is a major determinant of mortality outcomes post-AIS. We were also unable to assess the relationship between anemia severity and stroke severity. Fifth, despite higher rates of smoking among the polycythemia group, their outcomes were not worse than those observed in the non-anemic patients. Patients with polycythemia were significantly younger compared to other groups, suggesting that age-related factors may not have been fully accounted for in the multivariate model. Sixth, this study evaluated only all-cause mortality without cause-specific mortality data. Hence, it remains unclear whether the observed excess mortality among patients with anemia was attributable to stroke-related complications such as dysphagia with aspiration pneumonia, impaired nutrition, reduced mobility, falls or prolonged functional dependence, vs. competing mortality risks related to the underlying etiology of anemia. This distinction is particularly relevant for interpreting longer-term outcomes, as the relative contribution of the AIS event and baseline hemoglobin status may diminish over time, whilst the importance of comorbidity burden continues to increase.

## Conclusion

In this national registry study, anemia was associated with increased short- and long-term risks of all-cause mortality among patients with AIS compared to patients with normal hemoglobin levels. An elevated risk was observed even among those with mild anemia. The highest absolute risk was observed in patients with severe anemia, although no significant differences in mortality risk was observed among patients with polycythemia. Importantly, the excess risk associated with severe anemia appeared to attenuate by 10-years, whereas the greater risk with mild anemia persisted, underscoring the likely diminishing influence of baseline hemoglobin levels over long-term follow-up. In all, baseline hemoglobin level represents an important and potentially modifiable prognostic factor in AIS. Future studies should seek to define the hemoglobin thresholds that warrant transfusion, and determine whether acute or gradual correction improves outcomes in this patient population.

## Data Availability

The data used in this study are property of NRDO intended for internal usage. The deidentified data can be accessed for public health research purposes after approval from the Ministry of Health and Institutional Review Board.

## References

[B1] Adhami F. Liao G. Morozov Y. M. Schloemer A. Schmithorst V. J. Lorenz J. N. . (2006). Cerebral ischemia-hypoxia induces intravascular coagulation and autophagy. Am. J. Pathol. 169, 566–583. doi: 10.2353/ajpath.2006.051066 16877357 PMC1780162

[B2] Allport L. E. Parsons M. W. Butcher K. S. MacGregor L. Desmond P. M. Tress B. M. . (2005). Elevated hematocrit is associated with reduced reperfusion and tissue survival in acute stroke. Neurology 8:9. doi: 10.1212/01.wnl.0000183057.96792.a8 16275824

[B3] Barlas R. S. Honney K. Loke Y. K. McCall S. J. Bettencourt-Silva J. H. Clark A. B. . (2016). Impact of hemoglobin levels and anemia on mortality in acute stroke: analysis of UK regional registry data, systematic review, and meta-analysis. J. Am. Heart Assoc. 5:3019. doi: 10.1161/JAHA.115.003019 PMC5015269 27534421

[B4] Bateman G. A. Bateman A. R. (2024). Anemia in idiopathic intracranial hypertension increases cerebral blood flow and the intracranial pressure. J. Neuroophthalmol 44, 282–283. doi: 10.1097/WNO.0000000000001977 37669290

[B5] Bhatia R. S. Garg R. K. Gaur S. P. Kar A. M. Shukla R. Agarwal A. . (2004). Predictive value of routine hematological and biochemical parameters on 30-day fatality in acute stroke. Neurol. India 52, 220–223. 15269476

[B6] Braïk R. Jebali S. Blot P. L. Egbeola J. James A. Constatntin J. M. (2024). Liberal versus restrictive transfusion strategies in acute myocardial infarction: a systematic review and comparative frequentist and Bayesian meta-analysis of randomized controlled trials. Ann. Intensive Care 14:150. doi: 10.1186/s13613-024-01376-1 39340600 PMC11438751

[B7] Chohan S. A. How C. H. (2019). Long-term complications of stroke and secondary prevention: an overview for primary care physicians. Singapore Med. J. 60, 616–620. doi: 10.11622/smedj.2019158 31889205 PMC7911065

[B8] Derham R. J. Buchan P. C. (1989). Haemorheological consequences of oestrogen and progestogen therapy. Eur. J. Obstet. Gynecol. Reprod. Biol. 32, 109–114. doi: 10.1016/0028-2243(89)90191-3 2673883

[B9] Ducrocq G. Gonzalez-Juanatey J. R. Puymirat E. Lemesle G. Cachanado M. Durand-Zaleski I. . (2021). Effect of a restrictive vs liberal blood transfusion strategy on major cardiovascular events among patients with acute myocardial infarction and anemia: the REALITY randomized clinical trial. JAMA 325, 552–560. doi: 10.1001/jama.2021.0135 33560322 PMC7873781

[B10] Feigin V. L. (2021). Global, regional, and national burden of stroke and its risk factors, 1990–2019: a systematic analysis for the Global Burden of Disease Study 2019. The Lancet Neurology 20, 795–820. doi: 10.1016/S1474-4422(21)00252-0 34487721 PMC8443449

[B11] Ferrucci L. Guralnik J. M. Woodman R. C. Bandinelli S. Lauretani F. Corsi A. M. . (2005). Proinflammatory state and circulating erythropoietin in persons with and without anemia. Am. J. Med. 118:1288. doi: 10.1016/j.amjmed.2005.06.039 16271918

[B12] Gaspar B. L. Sharma P. Das R. (2015). Anemia in malignancies: pathogenetic and diagnostic considerations. Hematology 20, 18–25. doi: 10.1179/1607845414Y.0000000161 24666207

[B13] Global Burden of Disease. (2023). The rising global burden of stroke. eClinicalMedicine 59:2028. doi: 10.1016/j.eclinm.2023.102028 PMC10225620 37256100

[B14] Hess S. Y. Owais A. Jefferds M. E. D. Young M. F. Cahill A. Rogers L. M. (2023). Accelerating action to reduce anemia: review of causes and risk factors and related data needs. Ann. N Y Acad. Sci. 1523, 11–23. doi: 10.1111/nyas.14985 36987993 PMC10918744

[B15] Inui R. Koge J. Tanaka K. Yoshimoto T. Shiozawa M. Abe S. . (2023). Detrimental effect of anemia after mechanical thrombectomy on functional outcome in patients with ischemic stroke. Front. Neurol. 14:9891. doi: 10.3389/fneur.2023.1299891 PMC10770243 38187149

[B16] Kassebaum N. J. Jasrasaria R. Naghavi M. Wulf S. K. Johns N. Lozano R. . (2014). A systematic analysis of global anemia burden from 1990 to 2010. Blood 123, 615–624. doi: 10.1182/blood-2013-06-508325 24297872 PMC3907750

[B17] Köseoglu F. D. Özlek B. (2024). Anemia and iron deficiency predict all-cause mortality in patients with heart failure and preserved ejection fraction: 6-year follow-up study. Diagnostics (Basel) 14:209. doi: 10.3390/diagnostics14020209 38248085 PMC10814779

[B18] Kubo S. Hosomi N. Hara N. Neshige S. Himeno T. Takeshima S. . (2017). Ischemic stroke mortality is more strongly associated with anemia on admission than with underweight status. J. Stroke Cerebrovasc. Dis. 26, 1369–1374. doi: 10.1016/j.jstrokecerebrovasdis.2017.02.016 28256417

[B19] Maguida G. Shuaib A. (2023). Collateral circulation in ischemic stroke: an updated review. J Stroke 25, 179–198. doi: 10.5853/jos.2022.02936 36907186 PMC10250877

[B20] McLaren A. T. Marsden P. A. Mazer C. D. Baker A. J. Stewart D. J. Tsui A. K. . (2007). Increased expression of HIF-1alpha, nNOS, and VEGF in the cerebral cortex of anemic rats. Am. J. Physiol. Regul. Integr. Comp. Physiol. 292, R403–414. doi: 10.1152/ajpregu.00403.2006 16973934

[B21] Nagel T. Resnick N. Atkinson W. J. Dewey C. F. Gimbrone M. A. (1994). Shear stress selectively upregulates intercellular adhesion molecule-1 expression in cultured human vascular endothelial cells. J. Clin. Invest. 94, 885–891. doi: 10.1172/JCI117410 7518844 PMC296171

[B22] Ookawara S. Ito K. Sasabuchi Y. Hayasaka H. Kofuji M. Uchida T. . (2020). Associations of cerebral oxygenation with hemoglobin levels evaluated by near-infrared spectroscopy in hemodialysis patients. PLoS One 10:8. doi: 10.1371/journal.pone.0236720 PMC7416957 32776946

[B23] Portolés J. Martín L. Broseta J. J. Cases A. (2021). Anemia in chronic kidney disease: from pathophysiology and current treatments, to future agents. Front. Med. (Lausanne) 26:2296. doi: 10.3389/fmed.2021.642296 PMC8032930 33842503

[B24] Powers W. J. Rabinstein A. A. Ackerson T. Adeoye O. M. Bambakidis N. C. Becker K. . (2019). Guidelines for the early management of patients with acute ischemic stroke: 2019 update to the 2018 guidelines for the early management of acute ischemic stroke: a guideline for healthcare professionals from the American Heart Association/American Stroke Association. Stroke 50, e344–e418.030. doi: 10.1161/STR.0000000000000211 31662037

[B25] Ramos J. N. Calvão-Pires P. Gil I. Baptista T. Branco C. Branco G. . (2024). Hemoglobin in large vessel occlusion: look further than collaterals. J. Clin. Neurosci. 121, 100–104. doi: 10.1016/j.jocn.2024.02.010 38382284

[B26] Scheel K. W. (1990). “Development of coronary collaterals,” in: Coronary Circulation, eds. F. Kajiya, G. A. Klassen, J. A. E. Spaan, and J. I. E. Hoffman. Tokyo: Springer. doi: 10.1007/978-4-431-68087-1_20

[B27] Sharma K. Johnson D. J. Johnson B. Frank S. M. Stevens R. D. (2018). Hemoglobin concentration does not impact 3-month outcome following acute ischemic stroke. BMC Neurol 18:78. doi: 10.1186/s12883-018-1082-8 29859542 PMC5985053

[B28] Sico J. J. Myers L. J. Fenton B. J. Concato J. Williams L. S. Bravata D. M. . (2018). Association between admission haematocrit and mortality among men with acute ischaemic stroke. Stroke Vasc. Neurol. 3, 160–168. doi: 10.1136/svn-2018-000149 30294472 PMC6169611

[B29] Taccone F. S. Rynkowski C. B. Møller K. Lormans P. Quintana-Díaz M. Caricato A. . (2024). Restrictive vs liberal transfusion strategy in patients with acute brain injury: the TRAIN randomized clinical trial. JAMA 332, 1623–1633. doi: 10.1001/jama.2024.20424 39382241 PMC11581574

[B30] Tanne D. Molshatzki N. Merzeliak O. Tsabari R. Toashi M. Schwammenthal Y. . (2010). Anemia status, hemoglobin concentration and outcome after acute stroke: a cohort study. BMC Neurol. 10:22. doi: 10.1186/1471-2377-10-22 20380729 PMC2858127

[B31] Tefferi A. Barbui T. (2023). Polycythemia vera: 2024 update on diagnosis, risk-stratification, and management. Am. J. Hematol. 98, 1465–1487. doi: 10.1002/ajh.27002 37357958

[B32] Tian M. Li Y. Wang X. Tian X. Pei L. L. Wang X. . (2021). The hemoglobin, albumin, lymphocyte, and platelet (HALP) score is associated with poor outcome of acute ischemic stroke. Front. Neurol. 11:318. doi: 10.3389/fneur.2020.610318 PMC7835486 33510706

[B33] Turgeon A. F. Fergusson D. A. Clayton L. Patton M. P. Neveu X. Walsh T. S. . (2024). Liberal or restrictive transfusion strategy in patients with traumatic brain injury. N. Engl. J. Med. 391, 722–735. doi: 10.1056/NEJMoa2404360 38869931

[B34] Umit T. B. Akdogan H. I. Taskin Y. Yavuz Z. Sogut O. Arslan M. . (2026). Prognostic significance of the HALP score in patients with acute ischemic stroke. Postgrad. Med. J. 102, 283–289. doi: 10.1093/postmj/qgaf225 41430527

[B35] Venketasubramanian N. Chang H. M. Chan B. P. Young S. H. Kong K. H. Tang K. F. . (2015). Countrywide stroke incidence, subtypes, management and outcome in a multiethnic Asian population: the Singapore Stroke Registry–methodology. Int. J. Stroke 10, 767–769. doi: 10.1111/ijs.12472 25753306

[B36] Wester A. Attar R. Mohammad M. A. Andell P. Hofmann R. Jensen J. . (2019). Impact of baseline anemia in patients with acute coronary syndromes undergoing percutaneous coronary intervention: a prespecified analysis from the validate-swedeheart trial. J. Am. Heart Assoc. 8:2741. doi: 10.1161/JAHA.119.012741 PMC6759912 31387441

[B37] WHO. (2011). Hemoglobin, Concentrations for the Diagnosis of Anaemia and Assessment of Severity. Vitamin and Mineral Nutrition Information System. Geneva: WHO (WHO/NMH/NHD/MNM/11.1). Available online at: http://www.who.int/vmnis/indicators/hemoglobin (Accessed December 12, 2026).

[B38] Yan E. B. Hellewell S. C. Bellander B. M. Agyapomaa D. A. Morganti-Kossmann M. C. (2011). Post-traumatic hypoxia exacerbates neurological deficit, neuroinflammation and cerebral metabolism in rats with diffuse traumatic brain injury. J. Neuroinflammation 28:147. doi: 10.1186/1742-2094-8-147 PMC3215944 22034986

[B39] Zaid M. Sala L. Despins L. Heise D. Popescu M. Skubic M. . (2023). Cardiovascular sex-differences: insights via physiology-based modeling and potential for noninvasive sensing via ballistocardiography. Front. Cardiovasc. Med. 10:5958. doi: 10.3389/fcvm.2023.1215958 PMC10587415 37868782

