# Supplementary material for: Anemia impacts short-term and long-term mortality risk after acute ischemic stroke
Source: Front Stroke. 2026 Jul 28;5:1859301. doi: 10.3389/fstro.2026.1859301 (PMC13457158; doi:10.3389/fstro.2026.1859301)
Supplement: Supplementary file 1 [file Table_1.docx]

Supplementary Table 1: Other variables associated with all-cause mortality from 30-days to 10-years in the multivariable analysis

|  | **30-days (N = 66,614)** | | **1-year (N = 61,925)** | | **5-year (N = 32,257)** | | **10-year (N = 12,945)** | |
| --- | --- | --- | --- | --- | --- | --- | --- | --- |
| **Characteristic** | **HR** | **95% CI** | **HR** | **95% CI** | **HR** | **95% CI** | **HR** | **95% CI** |
| Hemoglobin Concentration |  |  |  |  |  |  |  |  |
| No anemia | * | | | | | | | |
| Mild anemia | 1.38 | 1.34, 1.42 | 1.33 | 1.29, 1.38 | 1.28 | 1.21, 1.35 | 1.16 | 1.04, 1.31 |
| Severe anemia | 1.99 | 1.92, 2.06 | 1.73 | 1.66, 1.81 | 1.52 | 1.41, 1.64 | 1.13 | 0.95, 1.34 |
| Polycythemia | 1.01 | 0.94, 1.08 | 1.00 | 0.93, 1.08 | 1.05 | 0.96, 1.16 | 1.06 | 0.90, 1.25 |
| GENDER |  |  |  |  |  |  |  |  |
| F | * | | | | | | | |
| M | 1.15 | 1.11, 1.18 | 1.17 | 1.13, 1.20 | 1.17 | 1.12, 1.23 | 1.18 | 1.08, 1.29 |
| ETHNIC GROUP |  |  |  |  |  |  |  |  |
| Chinese | * | | | | | | | |
| Indian | 1.06 | 1.01, 1.12 | 1.08 | 1.02, 1.14 | 0.98 | 0.90, 1.06 | 0.86 | 0.73, 1.00 |
| Malay | 1.28 | 1.23, 1.33 | 1.26 | 1.21, 1.31 | 1.20 | 1.14, 1.28 | 1.07 | 0.95, 1.20 |
| Others | 1.02 | 0.93, 1.12 | 0.98 | 0.88, 1.09 | 0.99 | 0.85, 1.15 | 0.95 | 0.73, 1.25 |
| AGE | 1.06 | 1.06, 1.07 | 1.07 | 1.07, 1.07 | 1.07 | 1.07, 1.07 | 0.86 | 0.70, 1.06 |
| HIST_SMOKING |  |  |  |  |  |  |  |  |
| No | * | | | | | | | |
| Yes | 1.23 | 1.19, 1.27 | 1.27 | 1.22, 1.31 | 1.26 | 1.20, 1.32 | 1.32 | 1.10, 1.58 |
| DIABETES MELLITUS |  |  |  |  |  |  |  |  |
| No | * | | | | | | | |
| Yes | 1.52 | 1.46, 1.58 | 1.53 | 1.47, 1.60 | 1.55 | 1.46, 1.64 | 1.36 | 1.23, 1.51 |
| HYPERLIPIDAEMIA |  |  |  |  |  |  |  |  |
| No | * | | | | | | | |
| Yes | 0.74 | 0.69, 0.79 | 0.78 | 0.72, 0.84 | 0.84 | 0.75, 0.93 | 0.92 | 0.79, 1.06 |
| HYPERTENSION |  |  |  |  |  |  |  |  |
| No | * | | | | | | | |
| Yes | 1.06 | 1.01, 1.12 | 1.07 | 1.01, 1.14 | 1.11 | 1.03, 1.20 | 1.03 | 0.87, 1.21 |
| ATRIAL FIBRILLATION |  |  |  |  |  |  |  |  |
| No | * | | | | | | | |
| Yes | 1.51 | 1.43, 1.60 | 1.43 | 1.35, 1.52 | 1.39 | 1.27, 1.52 | 1.26 | 1.10, 1.44 |
| PREVIOUS STROKE/TIA |  |  |  |  |  |  |  |  |
| No | * | | | | | | | |
| Yes | 1.05 | 0.97, 1.13 | 1.05 | 0.97, 1.14 | 1.06 | 0.95, 1.19 | 0.86 | 0.70, 1.06 |
| ISCHEMIC HEART DISEASE |  |  |  |  |  |  |  |  |
| No | * | | | | | | | |
| Yes | 1.43 | 1.36, 1.50 | 1.43 | 1.36, 1.50 | 1.36 | 1.27, 1.45 | 1.24 | 1.11, 1.38 |

HR = Hazard Ratio, CI = Confidence Interval

Adjusted for admission age + demographics and cardiovascular risk factors, specifically sex, race, hypertension, diabetes, hyperlipidemia, atrial fibrillation, previous stroke/TIA, ischemic heart disease and smoking status
